# Deubiquitinase Ubp3 regulates ribophagy and deubiquitinates Smo1 for appressorium‐mediated infection by *Magnaporthe oryzae*

**DOI:** 10.1111/mpp.13196

**Published:** 2022-02-27

**Authors:** Xuan Cai, Shikun Xiang, Wenhui He, Mengxi Tang, Shimei Zhang, Deng Chen, Xinrong Zhang, Caiyun Liu, Guotian Li, Junjie Xing, Yunfeng Li, Xiao‐Lin Chen, Yanfang Nie

**Affiliations:** ^1^ Laboratory of Physiological Plant Pathology South China Agricultural University Guangzhou China; ^2^ State Key Laboratory of Agricultural Microbiology and Provincial Key Laboratory of Plant Pathology of Hubei Province College of Plant Science and Technology Huazhong Agricultural University Wuhan China; ^3^ State Key Laboratory of Hybrid Rice Hunan Hybrid Rice Research Center Changsha China; ^4^ College of Materials and Energy South China Agricultural University Guangzhou China

**Keywords:** appressorium formation, deubiquitinating enzyme, Ras signalling, ribophagy, ubiquitination

## Abstract

The Ubp family of deubiquitinating enzymes has been found to play important roles in plant‐pathogenic fungi, but their regulatory mechanisms are still largely unknown. In this study, we revealed the regulatory mechanism of the deubiquitinating enzyme Ubp3 during the infection process of *Magnaporthe oryzae*. A*UBP3* deletion mutant was severely defective in appressorium turgor accumulation, leading to the impairment of appressorial penetration. During appressorium formation, the mutant was also defective in glycogen and lipid metabolism. Interestingly, we found that nitrogen starvation and rapamycin treatment induced the ribophagy process in *M. oryzae*, which is closely dependent on Ubp3. In the ∆*ubp3* mutant, the ribosome proteins and rRNAs were not well degraded on nitrogen starvation and rapamycin treatment. We also found that Ubp3 interacted with the GTPase‐activating protein Smo1 and regulated its de‐ubiquitination. Ubp3‐dependent de‐ubiquitination of Smo1 may be required for Smo1 to coordinate Ras signalling. Taken together, our results showed at least two roles of Ubp3 in *M. oryzae*: it regulates the ribophagy process and it regulates de‐ubiquitination of GTPase‐activating protein Smo1 for appressorium‐mediated infection.

## Graphical abstract

The deubiquitinating enzyme Ubp3 of *Magnaporthe oryzae* regulates the ribophagy process and de‐ubiquitinates GTPase‐activating protein Smo1 for appressorium‐mediated infection.

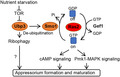

## 1 INTRODUCTION

Ubiquitination is a reversible posttranslational modification that regulates different cellular processes. The de‐ubiquitination process is catalysed by de‐ubiquitinating enzymes (DUBs) (Komander et al., 2009), of which ubiquitin‐specific proteases (UBPs) constitute the largest subfamily. There are 16 UBP genes in *Saccharomyces cerevisiae* and 27 UBP genes in *Arabidopsis thaliana* (Liu et al., 2008; Wilkinson, 1997). Our previous study identified 11 UBP genes in the rice blast fungus *Magnaporthe oryzae* and revealed that many of these are involved in growth, development, stress responses, nutrient utilization, and pathogenicity (Cai et al., 2020). In *M. oryzae*, the MoUbp14‐mediated de‐ubiquitination process is required for pleiotropic regulation during development and pathogenicity, and in particular MoUbp14 can regulate gluconeogenesis through de‐ubiquitinating Fbp1 and Pck1 (Wang et al., 2018). MoUbp4 and MoUbp8 are also required for growth, sporulation, infection‐related morphogenesis and pathogenicity, as well as detoxification of host stresses (Que et al., 2020; Yang et al., 2020). MoUbp8 is likely to be involved in the MoCreA‐mediated carbon catabolite repression (CCR) regulation system (Yang et al., 2020). However, the detailed regulatory mechanism by which UBPs regulate infection by *M. oryzae* remains unclear.

In yeast, ribosomes are rapidly degraded through a selective autophagy mechanism during nitrogen starvation, which is called “ribophagy” (Kraft et al., 2008). Studies have indicated that there is a link between ribophagy and ubiquitination (Bassham & MacIntosh, 2017; Kraft et al., 2008). Ubiquitination of ribosomal subunits or associated proteins may protect mature ribosomes from degradation by a selective autophagy pathway. During starvation, a de‐ubiquitination step promoted by Ubp3p/Bre5p may be required for the engulfment of ribosomal subunits by autophagic membranes or the maturation and/or fusion of the autophagosome with the vacuole (Kraft et al., 2008). The autophagy and proteasomal pathways also degrade distinct translation and RNA turnover factors in a Ubp3p‐dependent manner during nitrogen starvation (Kelly & Bedwell, 2015).

Ras proteins are highly conserved, small GTPases that act as a molecular switch and play an important role in many biological processes, including cell growth and differentiation (Milburn et al., 1990). Ras proteins cycle between an inactive GDP‐bound (Ras‐GDP) conformation and an active GTP‐bound (Ras‐GTP) conformation (Bos et al., 2007). Guanine nucleotide exchange factors (GEFs) and GTPase‐activating proteins (GAPs) switch the GTPase on and off competitively (Boguski & McCormick, 1993). There are two Ras proteins (Ras1 and Ras2) in *S*. *cerevisiae*, which are both essential for growth and function to activate adenylate cyclase (Tamanoi, 2011). Notably, studies have reported that RasGAP in yeast (Ira2) and humans (neurofibromin) are regulated by ubiquitination (Cichowski et al., 2003; Phan et al., 2010), and appropriate de‐ubiquitination of RasGAP by Ubp3 is important for Ras signalling (Li & Wang, 2013).

In *M. oryzae RAS2* is thought to be an essential gene that functions upstream of both the cAMP and Pmk1 signalling pathways in (Zhou et al., 2014). A wild‐type strain expressing the dominant active allele of *MoRAS2* (*MoRAS2* ^G18V^) showed abnormal appressoria and was nonpathogenic (Zhou et al., 2014). In this study, we set out to reveal the regulatory mechanism of the de‐ubiquitinating enzyme MoUbp3 in *M. oryzae*. We found that MoUbp3 plays key roles in appressorium‐mediated infection by regulating the ribophagy process and fine‐tunes the Ras‐mediated signalling pathway through de‐ubiquitinating the GTPase‐activating protein Smo1.

## 2 RESULTS

### 2.1 *UBP3* regulates vegetative growth and conidial formation of *M. oryzae*

**FIGURE 1:**
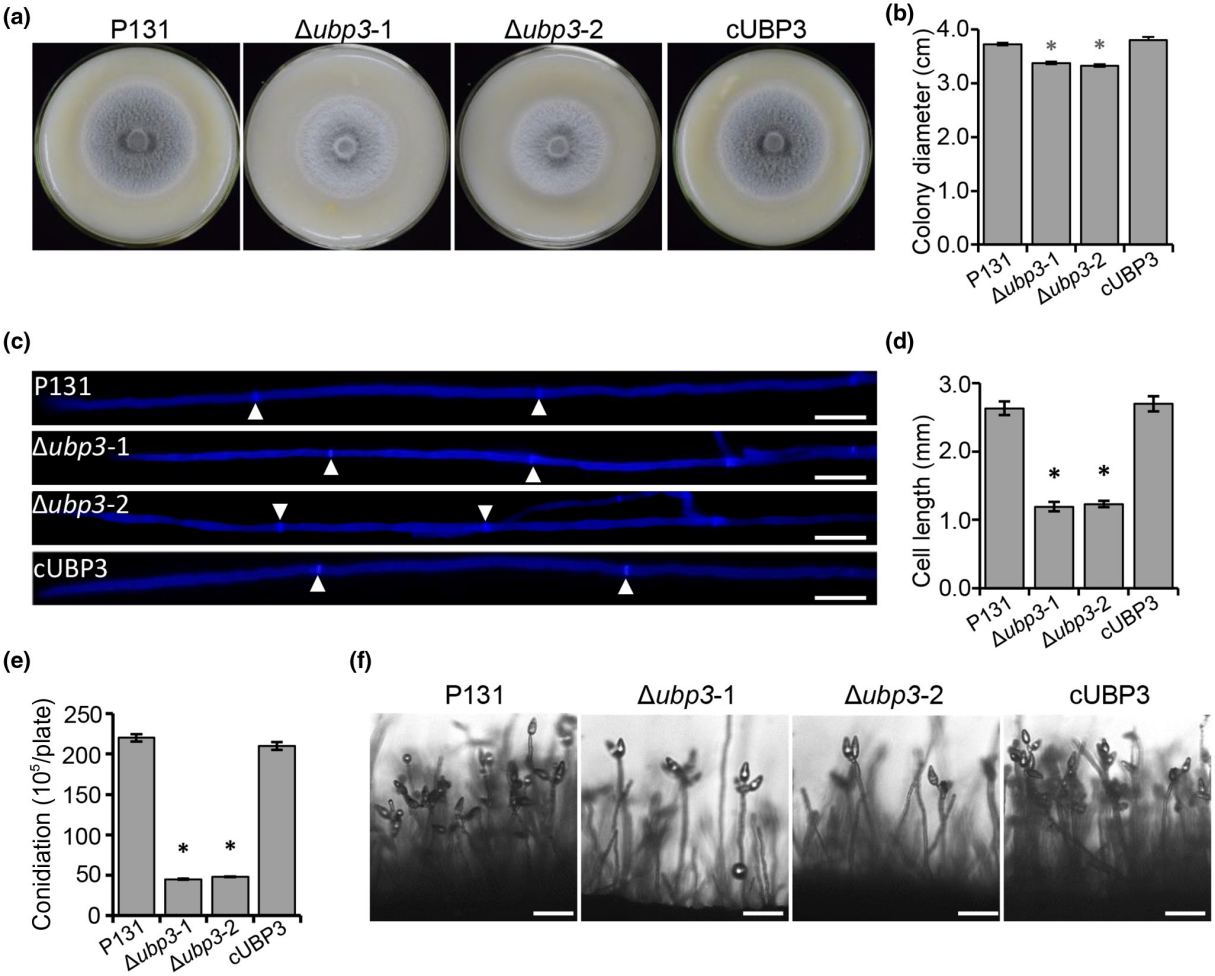
*UBP3* regulates vegetative growth and conidial formation of *Magnaporthe oryzae*. (a) Colony morphology of the wild‐type P131, two Δ*ubp3* mutants, and complementary strain cUBP3 were observed on oatmeal tomato agar (OTA) plates at 28℃ for 5 days. (b) The colony diameters of different strains were measured and subjected to statistical analysis. Significant differences compared with the wild type are indicated by an asterisk (*p* < 0.05). (c) Calcofluor white staining of hyphal tips shows the distance between septa in the subapical cell. White arrows indicate the cell septa. Bar, 10 μm. (d) Average cell length of the subapical cells in hyphal tips. Significant differences compared with the wild type are indicated by an asterisk (*p* < 0.05). (e) Statistical analysis of conidiation of the wild‐type P131, two Δ*ubp3* mutants, and cUBP3 strains. Error bars represent the standard deviation and asterisks represent significant difference (*p* < 0.05). (f) Conidial and conidiophore formation observed under a light microscope. Bar, 50 µm

To determine whether *UBP3* is related to vegetative growth in *M. oryzae*, we observed the colony morphology of Δ*ubp3* mutants, the wild‐type P131, and the complementary strain cUBP3 (Table S1), which were cultured on oatmeal tomato agar (OTA) plates for 5 days at 28°C. The colony sizes of the mutants were slightly reduced compared to the wild type (Figure 1a,b). The hyphal tip morphology of the Δ*ubp3* mutants was examined by staining with calcofluor white (CFW), and we found that the average length of subapical hyphal cells, which demonstrated a mature cell, was reduced compared to that of the wild type and the complementary strain (Figure 1c,d). These results indicate that *UBP3* is required for fungal vegetative growth. To investigate the roles of *UBP3* in conidium formation, we measured the conidiation capacity of the Δ*ubp3* mutants. Compared with the wild type and the complementary strain, conidiation of the Δ*ubp3* mutants was reduced by 79% (Figure 1e). The conidium formation on conidiophores of the Δ*ubp3* mutants was also reduced compared with the wild type and complementary strain (Figure 1f). These results indicate that *UBP3* is essential for vegetative growth and conidium formation.

### 2.2 *UBP3* is required for full virulence of *M. oryzae*

**FIGURE 2:**
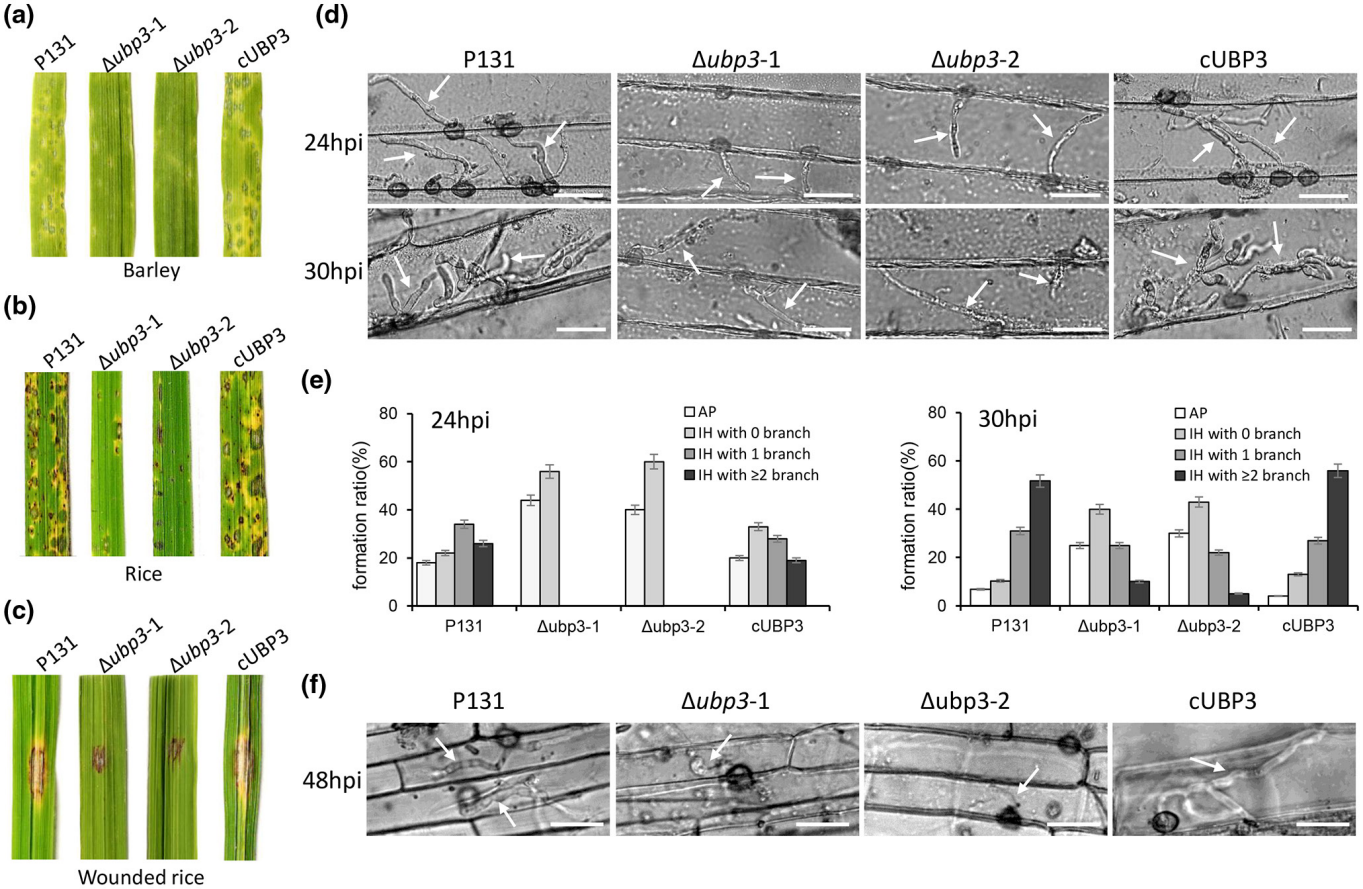
Deletion of *UBP3* leads to a reduction of virulence. Barley leaves (a) and rice leaves (b) were inoculated with conidial suspensions (10^5^ conidia/ml) of the wild‐type P131, Δ*ubp3*, and complementary strain cUBP3. Typical leaves were photographed at 5 days postinoculation (dpi). (c) Rice leaves were slightly wounded by a needle before inoculation with a mycelial block of different strains. Typical leaves were photographed at 5 dpi. (d) Colonization of different strains on barley epidermis at 24 and 30 h postinoculation (hpi). Arrows indicate the infection hypha. Bar, 25 µm. (e) Statistical analysis for different types of the infection hyphae (IH) at 24 and 30 hpi. Means and standard errors were calculated from three independent replicates (*n* > 100). (f) Colonization of different strains in rice leaf sheath cells at 48 hpi. Arrows indicate the infection hypha. Bar, 25 µm

To determine the effect of *UBP3* deletion on the pathogenicity of *M. oryzae*, conidial suspensions of the Δ*ubp3* mutants, the wild‐type P131, and complementary strain cUBP3 were sprayed onto susceptible rice seedlings and barley leaves. As shown in Figure 2a,b, very few lesions were formed on the rice seedlings and barley leaves inoculated with Δ*ubp3*. However, more and bigger lesions appeared on the leaves inoculated by P131 and cUBP3. When inoculating mycelial agar plugs onto wounded rice leaves, Δ*ubp3* mutants produced restricted spreading of the lesions (Figure 2c). These results indicate that *UBP3* plays an important role in full virulence of *M. oryzae*.

When observing the invasive growth on barley epidermis at 24 and 30 h postinoculation (hpi), we found that the infection hyphae (IH) formation of the Δ*ubp3* mutants was much slower than that of P131 and cUBP3. At 24 hpi, more than 80% of the appressoria of the wild type and complementary strains developed IH, but only around 60% of the appressoria of the Δ*ubp3* mutants formed IH. At 30 hpi, more than 80% of the wild‐type IH formed multiple branched IH, whereas no more than 40% of the Δ*ubp3* mutant IHs did so (Figure 2d,e). We also observed infection of the above strains in rice leaf sheath cells at 48 hpi; the Δ*ubp3* mutants were also significantly reduced in invasive growth (Figure 2f). Taken together, *UBP3* is required for invasive growth in barley host cells during infection.

### 2.3 Disruption of *UBP3* influences the formation and maturation of appressoria

**FIGURE 3:**
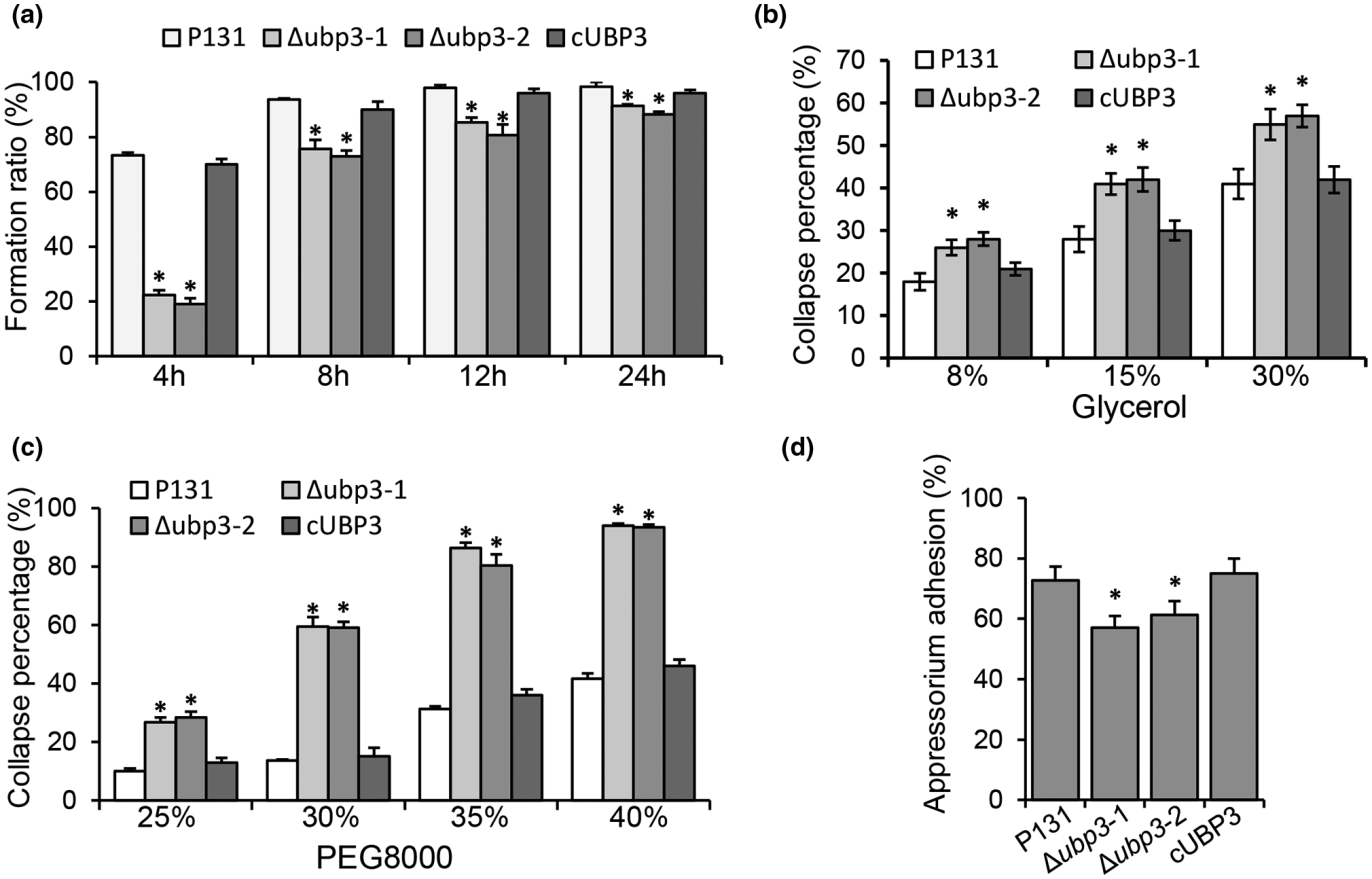
*UBP3* affects the appressorium development and adhesion. (a) Appressorium formation ratio of the wild‐type P131, Δ*ubp3*, and complementary strain cUBP3 at 24 h. Conidia were incubated on hydrophobic surfaces, and the appressorium formation ratio was calculated at different time points (4, 8, 12, and 24 h). Asterisks represent significant differences (*p* < 0.05, *n* > 100). (b) Collapse percentage of appressoria treated with glycerol. Mature appressoria on hydrophobic surfaces were treated with different concentrations of glycerol for 10 min before washing with water. Means and standard errors were calculated from three independent replicates (*n* > 100). Asterisks represent significant differences (*p* < 0.05). (c) Collapse percentage of appressoria treated with polyethylene glycol (PEG) 8000. Mature appressoria on hydrophobic surfaces were treated with different concentrations of PEG 8000 for 10 min before washing with water. Means and standard errors were calculated from three independent replicates (*n* > 100). Asterisks represent significant differences (*p* < 0.05). (d) Percentage of appressorial adhesion in different strains after 24 h. Means and standard errors were calculated from three independent replicates (*n* > 100). Asterisks represent significant differences (*p* < 0.05)

To understand why host penetration of the Δ*ubp3* mutants was blocked, we examined the formation and maturation of appressoria on hydrophobic coverslips. At 4 hpi only 20% of the conidia of Δ*ubp3* formed appressoria, while almost 70% of the conidia of P131 and cUBP3 formed appressoria. Compared with the wild type (more than 90%), the appressorium formation ratio of the mutants was significantly reduced to around 85% at 24 hpi (Figure 3a). These results indicate that *UBP3* is involved in appressorium formation.

As the Δ*ubp3* mutants formed dysfunctional appressoria for penetration, we used different concentrations of glycerol and polyethylene glycol (PEG) 8000 to treat the appressoria formed by different strains at 24 hpi to determine whether appressorial turgor of the Δ*ubp3* mutants was affected (Rocha et al., 2020). The result showed that, after 10 min of treatment, appressoria of the Δ*ubp3* mutants were more easily collapsed than those of the wild type and complementary strains with all tested concentrations of glycerol and PEG 8000 (Figure 3b,c), suggesting that the appressorial turgor of the Δ*ubp3* mutants was severely reduced. We also tested the appressorial adhesion of the above strains on a hydrophobic surface and found that the adhesion ability of the Δ*ubp3* mutants was reduced (Figure 3d). These results suggest that *UBP3* plays an important role in appressorium maturation and adhesion of *M. oryzae*.

### 2.4 ∆*ubp3* is defective in glycogen and lipid metabolism

**FIGURE 4:**
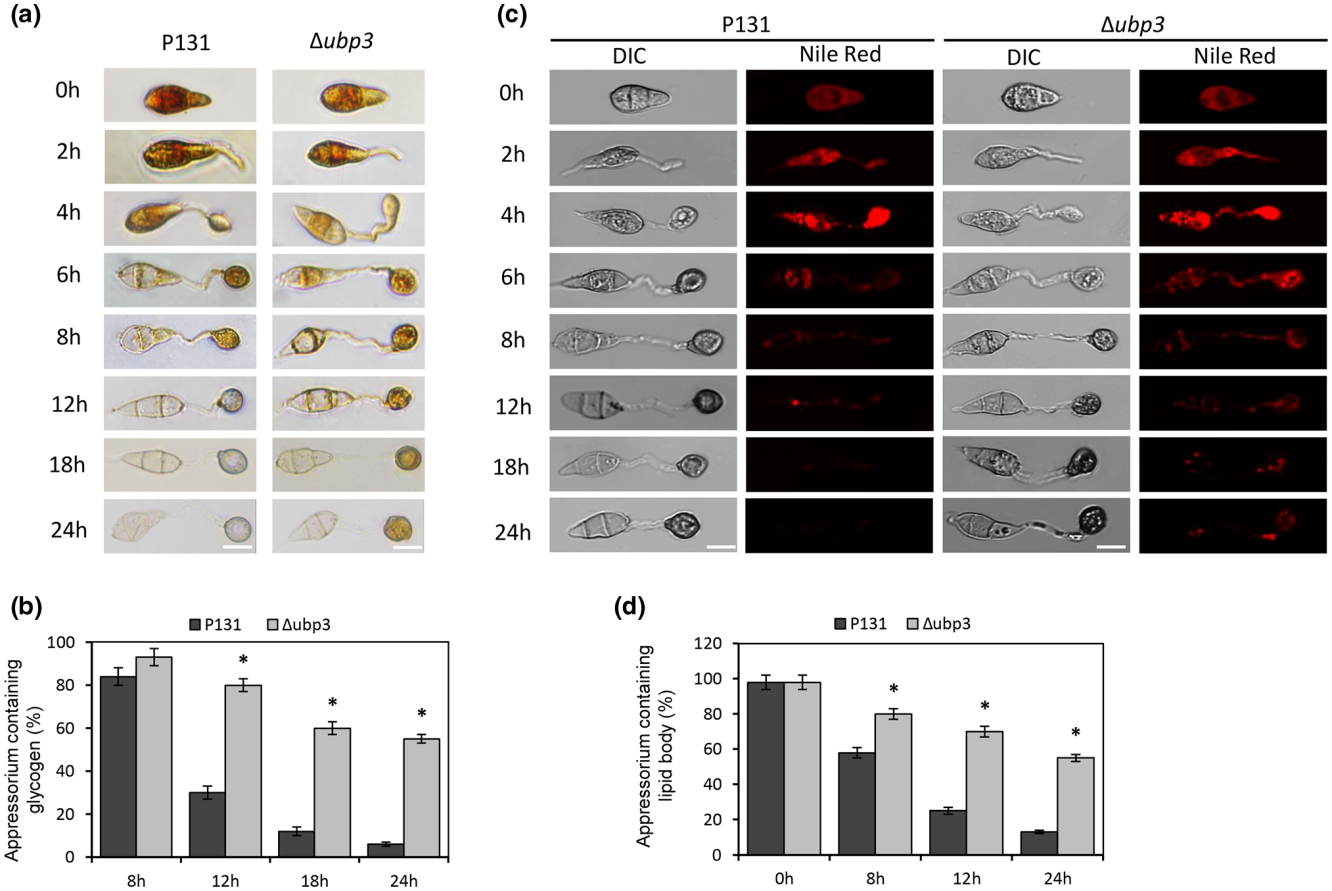
*UBP3* affects utilization of glycogen and lipid in appressoria. (a) Glycogen in conidia and appressoria of the wild‐type P131 and Δ*ubp3* was stained with KI/I_2_ for 0, 2, 4, 6, 8, 12, 18, and 24 h. Bar, 10 μm. (b) Quantification of the total glycogen content in appressoria in the indicated strains. Means and standard errors were calculated from three independent replicates. Asterisks indicate statistically significant differences (*p* < 0.05). (c) Lipids in conidia and appressoria of P131 and Δ*ubp3* were stained with Nile red for different times. Bar, 10 μm. (d) Quantitative analysis of lipid bodies during appressorium formation in the indicated strains. Means and standard errors were calculated from three independent replicates. Asterisks indicate statistically significant differences (*p* < 0.05)

The glycogen and lipids stored by conidia are important sources of nutrients for appressorium maturation. We used KI/I_2_ and Nile red to stain glycogen and lipids, respectively, to observe whether their movement and utilization were affected in the Δ*ubp3* mutant. The results showed that in wild‐type strain, glycogen began to gradually transfer from spores to appressoria at 8 hpi and almost disappeared at 12 hpi, while in the Δ*ubp3* mutant glycogen could still be observed at 18–24 hpi (Figure 4a,b), indicating that the metabolism of glycogen in the mutant was slowed down. Similar results were found with Nile red staining: lipid droplets began to be degraded at 8 h in the wild type and could not be detected at 12 hpi, while lipid droplets were still detected in the Δ*ubp3* mutant at 12–24 hpi (Figure 4c,d), indicating that the metabolism of lipid droplets in the mutant was also blocked. These results suggest that deletion of *UBP3* affects the metabolism of glycogen and lipids during appressorium maturation.

### 2.5 Ribophagy is found in *M. oryzae* and is involved in the TOR signalling pathway

**FIGURE 5:**
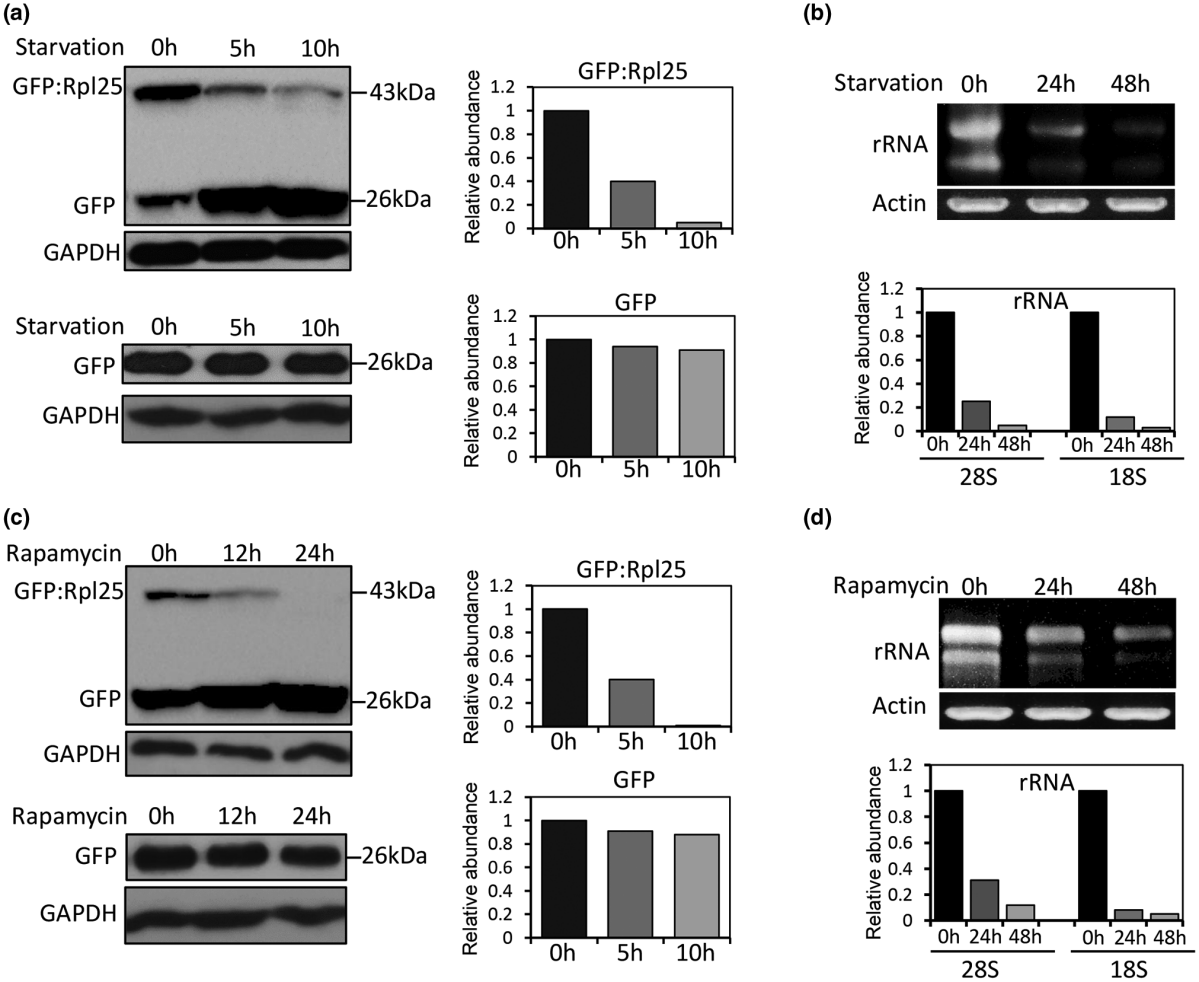
Ribophagy is found during nitrogen starvation and rapamycin treatment in *Magnaporthe oryzae*. (a) The spore suspensions of strains containing GFP:Rpl25 were cultured in liquid complete medium (CM) for 36 h and then transferred to nitrogen‐deficient minimal medium (MM−N) for 5 or 10 h. Western blots and quantification of Rpl25 from the wild‐type P131 (WT) harvested at various times following the onset of nitrogen starvation. The WT strain containing green fluorescent protein (GFP) was used as a control and GAPDH was used as an internal control. (b) RNA gel and quantification of rRNA. The spore suspensions of strains containing GFP:Rpl25 were cultured in liquid CM for 36 h and then transferred to MM−N for 24 or 48 h. RNA gel and quantification of rRNA from the wild‐type P131 harvested at various times following the onset of nitrogen starvation. The mRNA level of actin gene *ACT1* was used as an internal control. (c) Spore suspensions of strains containing GFP:Rpl25 were cultured in liquid CM for 36 h and then were treated with rapamycin for 12 or 24 h. Western blot and quantification of Rpl25 from the wild‐type P131 harvested at various times following treatment with 100 ng/ml rapamycin. The WT strain containing GFP was used as a control and GAPDH was used as an internal control. (d) Spore suspensions of strains containing GFP‐Rpl25 were cultured in liquid CM for 36 h and then were treated with rapamycin for 24 or 48 h. RNA gel and quantification of rRNA from the wild‐type P131 harvested at various times following treatment with 100 ng/ml rapamycin. The mRNA level of actin gene *ACT1* was used as an internal control

Studies in yeast have shown that nitrogen starvation can induce selective autophagy of ribosomes, called ribophagy, which has been found to depend on Ubp3p in *S. cerevisiae* (Kelly & Bedwell, 2015; Kraft et al., 2008; Ossareh‐Nazari et al., 2010). To investigate whether ribophagy also exists in *M. oryzae*, we monitored the abundance of the large ribosomal subunit protein Rpl25 and rRNAs during nitrogen starvation. The results showed that the abundance of Rpl25 and two rRNAs (28S and 18S) were significantly decreased under nitrogen starvation conditions for 5 and 10 h (Figure 5a,b), suggesting that nitrogen starvation indeed induced ribophagy in *M. oryzae*. Similar to nitrogen starvation, rapamycin, an inhibitor of the target of rapamycin (TOR) signalling pathway, also induced degradation of Rpl25 at 12 and 24 h (Figures 5c,d and S1).

**FIGURE 6:**
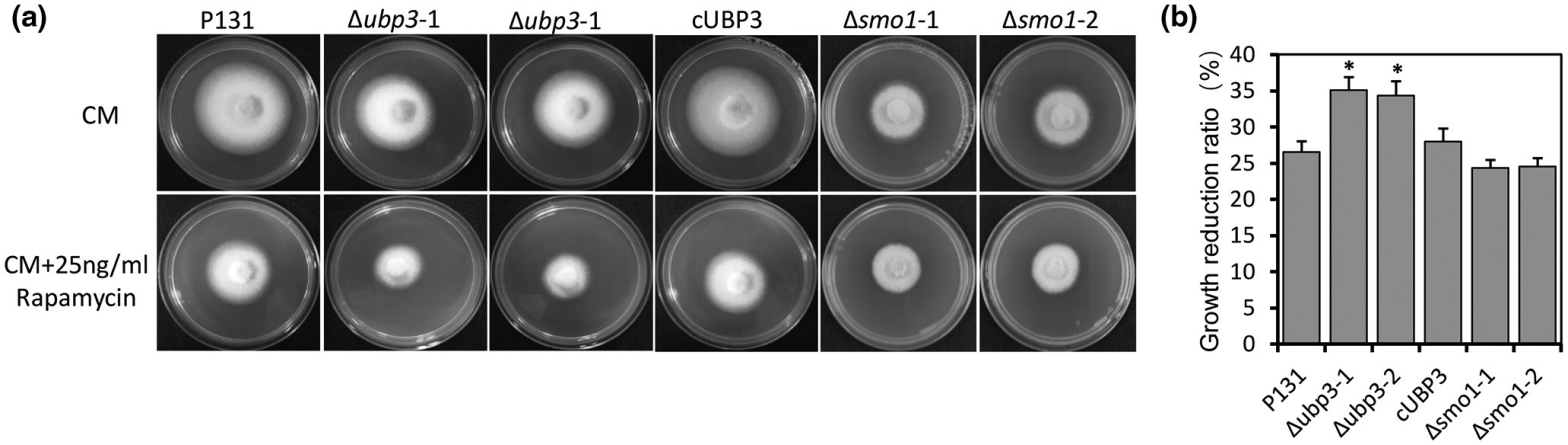
The Δ*ubp3* mutant is sensitive to rapamycin. (a) The wild‐type P131, the Δ*ubp3* mutants, complementary strain cUBP3, and the Δ*smo1* mutants were inoculated on fresh complete medium (CM) plates supplemented with 25 ng/ml rapamycin at 28℃ for 5 days. (b) Statistical analysis of inhibition rate based on colony diameters. Means and standard errors were calculated from three independent replicates. Significant differences are indicated by asterisks (*p* < 0.05)

To explore whether the Ubp3‐mediated ribophagy is relevant to the TOR signalling pathway, we cultured the Δ*ubp3* mutants, the wild‐type P131, and cUBP3 in medium supplemented with rapamycin. The results indicated that the Δ*ubp3* mutants were more sensitive to rapamycin (Figure 6a,b). Taken together, these results suggest that ribophagy induced by nitrogen starvation and rapamycin depends on Ubp3 in *M. oryzae*.

### 2.6 Degradation of ribosome subunit protein Rpl25 is regulated by Ubp3

**FIGURE 7:**
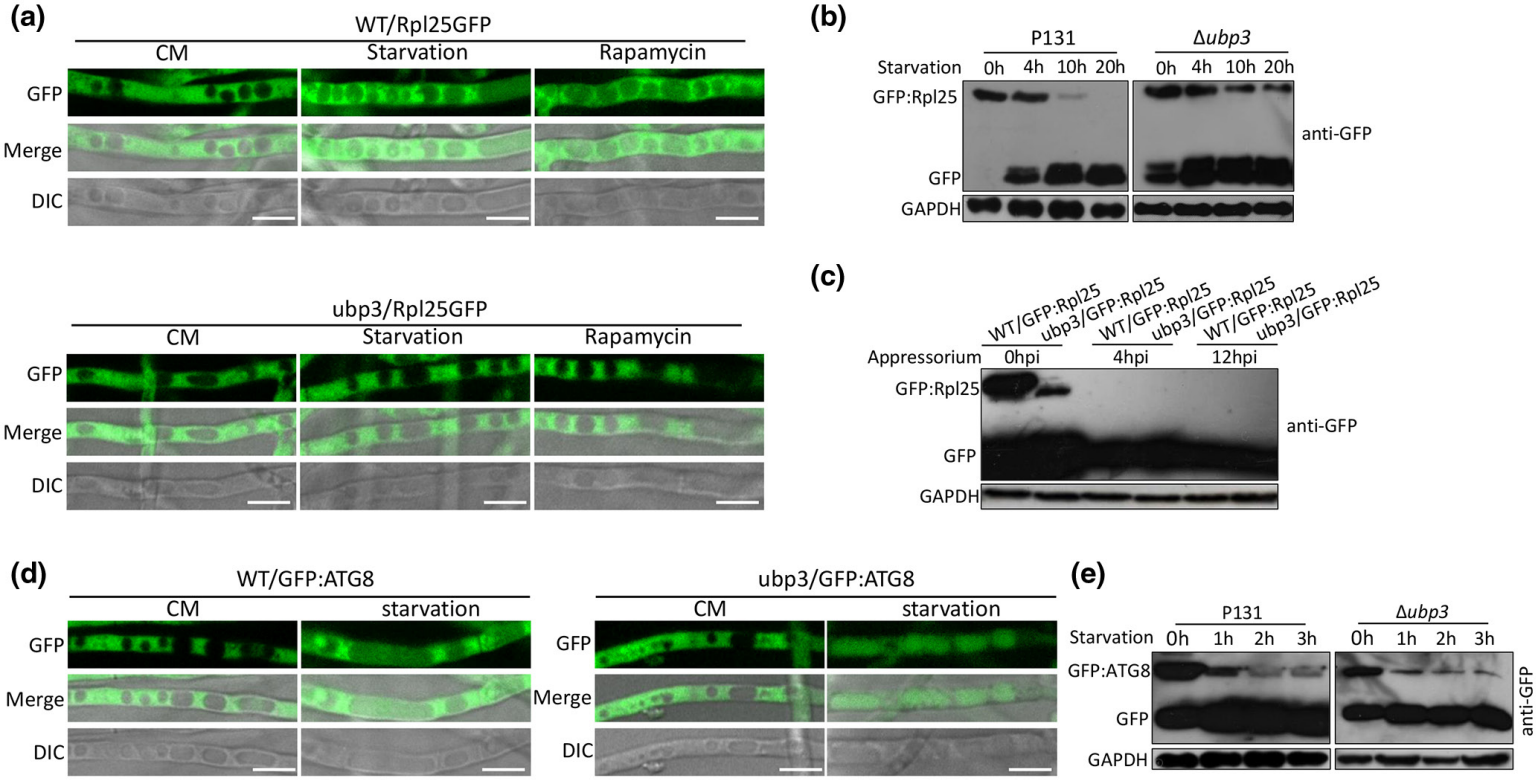
Ribophagy in *Magnaporthe oryzae* depends on *UBP3*. (a) Subcellular localization of GFP:Rpl25. Spore suspensions of strains expressing GFP:Rpl25 were cultured in complete medium (CM) for 36 h and then transferred to nitrogen‐deficient minimal medium (MM−N; starvation) or 25 ng/ml rapamycin treatment. The hyphae were examined by fluorescence microscopy. Bar, 10 μm. (b) Western blot of Rpl25 from the wild‐type P131 and Δ*ubp3* strains harvested at various times following the onset of nitrogen starvation. (c) Protein level of GFP:Rpl25 in P131 and Δ*ubp3* during appressorium formation stage. At 4 and 12 h postinoculation (hpi) during appressorium formation, free green fluorescent protein (GFP) but no GFP:Rpl25 band could be detected. GAPDH was used as an internal control. (d) Subcellular localization of GFP:ATG8. Spore suspensions of strains expressing GFP:ATG8 were cultured in rich medium for 36 h (before starvation) and then transferred to nitrogen‐deficient medium for 10 h (starvation). The hyphae were examined by fluorescence microscopy. Bar, 10 μm. (e) Western blots of ATG8 from the wild‐type P131 and Δ*ubp3* strains harvested at various times following the onset of nitrogen starvation

Because ribophagy requires ubiquitin protease Ubp3 in yeast, we then determined whether *UBP3* is required for ribophagy in *M. oryzae*. We transformed GFP‐Rpl25 fusion constructs into the wild type and the Δ*ubp3* mutant. After culturing in liquid complete medium (CM) for 36 h, mycelia of Δ*ubp3* and P131 were transferred into liquid minimal medium (MM) with reduced nitrogen (MM−N) or liquid CM with 25 ng/ml rapamycin. As shown in Figure 7a, GFP‐Rpl25 was distributed throughout the cytoplasm when cells were grown under rich conditions after 15–20 h of starvation and rapamycin treatment; however, the green fluorescent protein (GFP) signal accumulated in the vacuole in the wild‐type strain. In contrast, GFP:Rpl25 in most mycelia was still distributed in the cytoplasm of Δ*ubp3*. Total proteins were extracted from P131 and Δ*ubp3* expressing GFP:Rpl25 that were incubated for 0, 4, 10 and 20 h in MM−N medium. Free GFP and the fusion protein GFP:Rpl25 were then detected using the anti‐GFP antibody. The western blot results showed that, in the wild type, the abundance of GFP:Rpl25 was significantly reduced after 10 h of nitrogen starvation treatment; meanwhile, free GFP gradually accumulated (Figure 7b). However, in the Δ*ubp3* mutant, GFP:Rpl25 still existed after 20 h of starvation, which means that the degradation of Rpl25 was blocked. We also detected GFP:Rpl25 in P131 and Δ*ubp3* during the appressorium formation stage. At 4 and 12 hpi during appressorium formation, free GFP but no GFP:Rpl25 band could be detected, while GFP:Rpl25 could be detected from both of the strains WT/GFP:Rpl25 and ubp3/GFP:Rpl25 in the conidia sample (0 hpi) (Figure 7c). This result suggests that GFP:Rpl25 is degraded during appressorium formation, suggesting ribophagy occurs during appressorium formation.

In yeast Ubp3p can interact with chaperone‐like protein Cdc48p and ubiquitin‐binding adaptor Ufd3p to regulate ribophagy (Ossareh‐Nazari et al., 2010). Yeast Cdc48p may act as a molecular platform to facilitate the ribosomes deubiquitinated by Ubp3p (Ossareh‐Nazari et al., 2010). Therefore, we tested whether *M. oryzae* Ubp3 also interacts with these partner proteins. However, the yeast two‐hybrid assay suggested that both *M. oryzae* homologs of Cdc48p and Ufd3p did not interact with Ubp3 (Figure S2).

### 2.7 General autophagy is not affected in ∆*ubp3*

GFP‐ATG8 can be used to assay ATG8 flux into the vacuole (Nair et al., 2011). To determine whether the general autophagy in *M. oryzae* requires *UBP3*, we also transformed GFP:ATG8 fusion constructs into P131 and Δ*ubp3*. After nitrogen starvation treatment for 5 h, both P131 and Δ*ubp3* cells showed normal GFP:ATG8 transportation to the vacuole (Figure 7d). Western blotting of proteins extracted from P131 and Δ*ubp3* also showed the same result: the abundance of GFP:ATG8 gradually reduced after nitrogen starvation at 1, 2, and 3 h in both strains (Figure 7e). Therefore, general autophagy is not affected in ∆*ubp3*.

### 2.8 *UBP3* deletion mutant increased in total protein ubiquitination level

**FIGURE 8:**
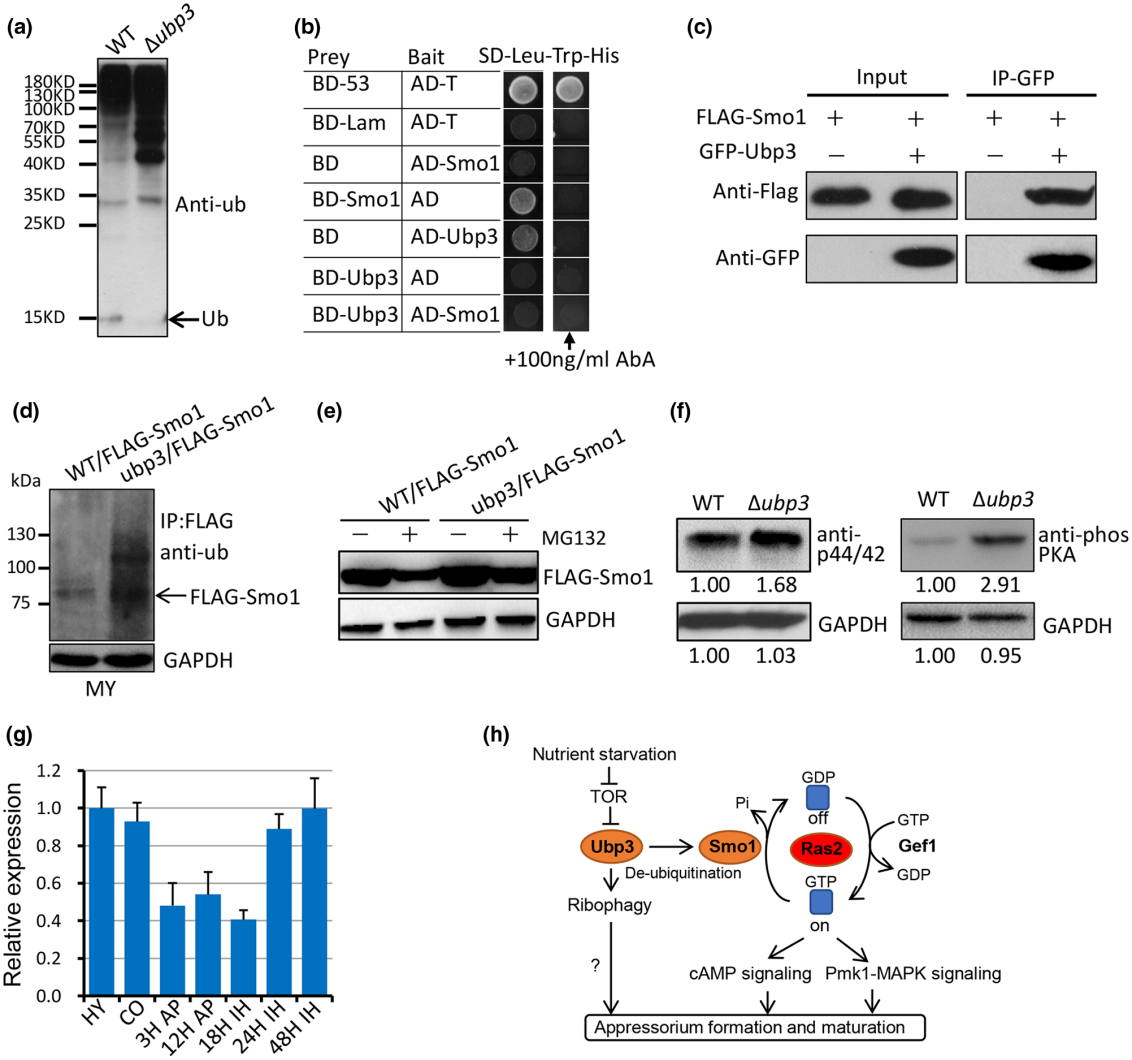
Ubp3 regulates the ubiquitination of Smo1. (a) *UBP3* deletion mutant increased in total protein ubiquitination level compared to the wild type (WT). (b) Yeast two‐hybrid analysis confirmed protein–protein interactions between Ubp3 and Smo1. Cultures of AH109 yeast cells containing plasmids encoding different combinations of prey (AD) and bait (BD) vectors were grown on selective (SD−Leu−Trp−His) plates and incubated at 30°C for 3 days. The vectors BD‐53/AD‐T and BD‐Lam/AD‐T were expressed in yeast as positive and negative controls, respectively. A cyclic depsipeptide antibiotic aureobasidin A (AbA) was added to the −Leu−Trp−His plate to inhibit autoactivation. *LacZ* autoactivation was observed when Smo1 was cloned on the BD vector and Ubp3 was cloned on the AD vector. (c) Co‐immunoprecipitation analyses between Ubp3 and Smo1. In proteins eluted from anti‐green fluorescent protein (GFP) beads, the FLAG:Smo1 band was also detected in the transformant expressing the GFP‐Ubp3 construct by anti‐FLAG antibody. (d) Ubiquitination level of Smo1 in the wild‐type P131 and Δ*ubp3* strains expressing FLAG‐Smo1. Total proteins were used in a pulldown assay with anti‐FLAG M2 beads and the ubiquitination level of Smo1 was detected with anti‐ubiquitin antibodies. (e) Protein level of FLAG:Smo1 in mycelia treated with or without MG132, a potent cell‐permeable proteasome inhibitor. (f) Phosphorylation level of mitogen‐activated protein kinases (MAPKs) and protein kinase A (PKA) in the wild‐type P131 and Δ*ubp3* strains. Total proteins were separated in a phospho‐tag gel by electrophoresis and western blotting was performed with anti‐p44/42 antibody or anti‐phosPKA antibody, respectively. (g) *UBP3* expression at stages of hyphae, conidia, appressoria, and infectious hyphae. HY, hyphae; CO, conidia; 3H AP, appressorium at 3 h; 12H AP, appressorium at 12 h; 18H IH, infectious hyphae at 18 h; 24H IH, infectious hyphae at 24 h; 48H IH, infectious hyphae at 48 h. (h) Regulatory model of Ubp3. Model for the potential regulatory mechanism of Ubp3 in de‐ubiquitinating Smo1 and in the Ras2 signalling pathway

Because *UBP3* is predicted to encode a de‐ubiquitinating enzyme, we compared the total ubiquitination levels of the wild type and the Δ*ubp3* mutant. Total proteins were extracted from mycelia incubated in liquid CM. Western blot analysis was performed by using an anti‐ubiquitin antibody to detect total ubiquitination levels. Compared with that of the wild type, the total ubiquitination level of the Δ*ubp3* mutant was significantly increased. Notably, the mono‐ubiquitin and poly‐ubiquitin bands accumulated more in the Δ*ubp3* mutant (Figure 8a), suggesting that *UBP3* is indeed involved in protein de‐ubiquitination.

### 2.9 Ubp3 interacts with Ras GTPase‐activating protein Smo1

Previous studies reported that Ubp3 interacts with GTPase‐activating proteins (RasGAP) Ira2 and regulates its ubiquitination in yeast (Li & Wang, 2013). In *M. oryzae*, Smo1 was identified as a RasGAP and is necessary for the regulation of RAS2 activation during infection‐related development (Kershaw et al., 2019). We wondered whether Smo1 can be de‐ubiquitinated by Ubp3 in *M. oryzae*. We first employed a yeast two‐hybrid assay to test whether Ubp3 directly interacts with Smo1 by constructing BD‐Ubp3 and AD‐Smo1. The results showed that Ubp3 did not directly interact with Smo1 (Figure 8b). To further examine whether Ubp3 interacted with Smo1 in *M. oryzae*, GFP‐Ubp3 and FLAG‐Smo1 fusion constructs were generated and introduced by co‐transformation into the wild‐type strain. Total protein was isolated from the positive transformants and subsequently co‐immunoprecipitation (Co‐IP) was performed. In proteins eluted from anti‐GFP beads, the FLAG:Smo1 band was also detected in the transformant expressing the GFP‐Ubp3 construct (Figure 8C). These results suggest that Ubp3 interacts with Smo1 in *M. oryzae*.

### 2.10 Ubp3 de‐ubiquitinates Smo1

To further determine whether Smo1 can be de‐ubiquitinated in *M. oryzae*, we transferred the FLAG‐Smo1 construct into P131 and Δ*ubp3*. The proteins extracted from the mycelial hyphae were purified by anti‐FLAG beads and then were analysed by western blotting with anti‐ubiquitin antibodies to reveal ubiquitinated FLAG‐Smo1. The results showed that, in comparison with the wild type, the ubiquitination level of Smo1 increased the Δ*ubp3* mutant (Figure 8d). However, when treated with the proteasome inhibitor MG132, the protein level of Smo1 in the Δ*ubp3* mutant was not increased but slightly reduced (Figure 8e), suggesting that ubiquitination of Smo1 is not involved in its degradation. To determine the effects of Smo1 de‐ubiquitination, we assessed the phosphorylation level of mitogen‐activated protein kinases (MAPKs) and protein kinase A (PKA) in the wild type and the Δ*ubp3* mutant using anti‐p44/42 and anti‐phospho‐PKA antibodies, respectively. We found that the phosphorylation level of MAPKs and PKA were both increased in the Δ*ubp3* mutant (Figure 8f). We also tested sensitivity of Δ*smo1* to rapamycin, but there were no significant changes compared to the wild type (Figure 6a,b). These data suggest that Ubp3 may function as a negative regulator to coordinate functional regulation of Ras proteins through de‐ubiquitinating Smo1 in *M. oryzae*. Expression profiling by reverse transcription quantitative real‐time PCR (RT‐qPCR) analysis showed that *UBP3* was highly expressed in mycelial hyphae, conidia, and invasive hyphae at 24 and 42 hpi, but less expressed during the appressorium formation stage at 3 and 12 hpi (Figure 8g), further confirming the hypothesis that Ubp3 may function as a negative regulator in the appressorium stage.

## 3 DISCUSSION

Studies have reported that ubiquitination plays important roles in regulating the abundance and function of proteins (Li et al., 2020; McCafferty & Talbot, 1998; Oh et al., 2012; Shi et al., 2016). De‐ubiquitination also plays important roles in the growth and pathogenicity of plant‐pathogenic fungi (Cai et al., 2020; Que et al., 2020; Wang et al., 2018; Yang et al., 2020). However, the regulatory mechanisms of DUBs remain largely unclear. In this study, we tried to explore the regulatory mechanism of the de‐ubiquitinase Ubp3 in *M. oryzae*. In this work, we provide evidence that Ubp3 is required for ribophagy under nitrogen starvation and also functions as a negative regulator of the Ras signalling pathway for appressorium formation and maturation during infection of *M. oryzae*.

Ribosome biogenesis and protein translation are among the most energy‐consuming cellular processes. In *S. cerevisiae*, ribosomes are rapidly degraded through a selective autophagy mechanism during nitrogen starvation, which is called ribophagy (Kraft et al., 2008). Here we show that the ribophagy process is also found in *M. oryzae*, which is Ubp3‐dependent and induced by nitrogen starvation and rapamycin. We found that ribophagy‐mediated degradation of the ribosome protein Rpl25 requires Ubp3. In yeast, Ubp3p can interact with Bre5p to form a Ubp3p/Bre5p complex, which can regulate different cellular processes, including transcription elongation and the protein kinase C signalling pathway (Kvint et al., 2008; Li et al., 2005, 2007; Wang et al., 2008). However, the detailed regulatory mechanism of *M. oryzae* Ubp3 during ribophagy requires further study.

We found that Ubp3‐mediated ribophagy is responsive to the TOR signalling pathway because deletion of *UBP3* resulted in an increased sensitivity to rapamycin and a block in ribophagy (Figures 5c and 6a). Previous studies in *S. cerevisiae* showed that TOR signalling is repressed during nitrogen starvation and is required for activating Ubp3p‐mediated ribophagy (Pestov & Shcherbik, 2012). The TOR signalling pathway is a central nutrient signalling pathway that works in parallel with the Ras/PKA signalling pathway and negatively regulates macro‐autophagic process during appressorium formation in *M. oryzae* (Marroquin‐Guzman et al., 2017; Marroquin‐Guzman & Wilson, 2015). Whether or not the TOR signalling‐responsive ribophagy process is required for function of appressoria needs further study.

There is an antagonistic relationship between the TOR and Ras/PKA signalling pathways. In other organisms, enhanced sensitivity to rapamycin often leads to hyperactivation of the Ras/PKA signalling pathway (Li & Wang, 2013; Ramachandran & Herman, 2011). The deletion mutant of *UBP3* was sensitive to rapamycin, which is evidence that the lack of de‐ubiquitination of Smo1 leads to the overactivation of RAS2 and the downstream PKA pathway. Ras proteins switch between active GTP and inactive GDP statuses, which regulate cellular responses to external stimuli by activating various downstream signalling pathways (Cherfils & Zeghouf, 2013). In plant‐pathogenic fungi, Ras signalling is very important for virulence (Bluhm et al., 2007; Fortwendel et al., 2004; Muller et al., 2003; Waugh et al., 2002). Ras2 functions upstream from both cAMP‐PKA signalling and the Kss1 MAPK pathways to regulate pseudohyphal growth in yeast and *M. oryzae* (Mösch et al., 1996; Tamanoi, 2011). *M. oryzae RAS2* is thought to be an essential gene and the wild‐type strain expressing the dominant active allele of MoRAS2 (*MoRAS2* ^G18V^) shows abnormal appressoria and is nonpathogenic. Expression of dominant active MoRas2 may overstimulate both the cAMP‐PKA signalling and Pmk1 MAPK signalling pathways (Zhou et al., 2014).

We found that Ubp3 interacted with Smo1 and regulated its ubiquitination level. Smo1 functions as a GTPase‐activating protein (RasGAP), together with guanine nucleotide exchange proteins (RasGEF), to regulate the balance of Ras proteins in *M. oryzae* (Kershaw et al., 2019). The Δ*smo1* strain shows defective conidial adhesion due to reduced spore tip mucilage. Like the Δ*ubp3* mutant, Δ*smo1* is also delayed in appressorium formation, reduced in turgor accumulation, and impaired in penetration and infection capacity to rice plants (Kershaw et al., 2019). Therefore, the Δ*ubp3* mutant mimics Δ*smo1*, reflecting a similar role of Ubp3 and Smo1 in *M. oryzae*.

The Ubp3‐mediated de‐ubiquitination of Smo1 therefore functions as a switch to control the Ras signalling pathway. During appressorium formation, Smo1 negatively regulates Ras by converting it from the GTP‐bound active to the GDP‐bound inactive form (Kershaw et al., 2019). Consistent with this, the *UBP3* gene is expressed at a low level during appressorium formation (Figure 8g); therefore, the de‐ubiquitination process of Smo1 is also low level, which triggers ubiquitination and inactivation of Smo1 and increases GTP‐bound active Ras proteins (such as Ras2). The resulting Ras‐GTP triggers the activation of the cAMP‐PKA signalling and Pmk1‐MAPK signalling pathways for appressorium formation and maturation (Figure 8h). A similar regulatory mechanism has been revealed in yeast (Li & Wang, 2013), indicating that Ubp3‐mediated de‐ubiquitination of RasGAP may function as a conserved mechanism to control Ras signalling. Given the crucial roles of Ras signalling in infection by *M. oryzae*, the de‐ubiquitinating enzyme Ubp3 may offer a new target for developing fungicides.

Besides ribophagy and the protein Smo1, in yeast some other functions have also been revealed. For example, by regulating the activity of assembly silent information regulator SIR4, Ubp3 can function as an inhibitor of silencing (Moazed & Johnson, 1996). Ubp3 can respond to external stimuli for transcriptional activation upon osmostress, which is regulated by the Hog1 stress‐activated protein kinases (SAPKs) (Solé et al., 2011). Also, Ubp3 mediates trafficking between the endoplasmic reticulum and Golgi bodies through de‐ubiquitinating the COPII and COPI proteins (Sec23p and Sec27p) (Cohen et al., 2003a, 2003b). It would be interesting to test whether *M. oryzae* Ubp3 is involved in the above‐mentioned or other processes.

## 4 EXPERIMENTAL PROCEDURES

### 4.1 Strains and culture conditions

P131 was used as the wild‐type strain of *M. oryzae* (Table S1) (Chen et al., 2014). The *UBP3* deletion mutants were obtained in our previous study (Cai et al., 2020). All strains were cultured at 28°C on OTA plates. Mycelia cultured in liquid CM (180 rpm) incubated at 28°C for 36 h were used to extract genomic DNA, RNA, protein, and isolating protoplasts. Colony growth and conidiation were performed as described previously (Chen et al., 2014). Conidia from 7‐day‐old OTA cultures were harvested for testing. For the rapamycin sensitivity assay, strains were cultured on the CM plates supplemented with 25 ng/ml rapamycin (Sigma‐Aldrich).

### 4.2 Infection assay

One‐month‐old rice seedlings (*Oryza sativa* ‘LTH’) and 1‐week‐old barley leaves (*Hordeum vulgare* ‘E9’) were used for virulence tests. The conidial suspension (5 × 10^4^ conidia/ml in 0.025% Tween 20) was used to spray plants, which then were incubated in full humidity conditions at 28°C for 5 days to examine the disease lesions.

The conidial suspension (10^5^ conidia/ml) was dropped onto a hydrophobic coverslip and incubated in a dark moist chamber at 28°C for 4, 8, 12, and 24 hpi to test the formation ratio of the wild‐type P131, Δ*ubp3* mutants, and cUBP3 under a microscope (Ni90; Nikon). Appressoria formed at 24 hpi on the hydrophobic coverslip were treated with different concentrations of glycerol (8%, 15%, and 30%, wt/vol) or PEG 8000 (25%, 30%, 35%, and 40%, wt/vol) for 10 min to calculate appressoria collapse ratios under a microscope.

### 4.3 Staining assays

Mycelium was stained with 10 µg/ml CFW for 5 min and observed under a fluorescence microscope (Ni90 ; Nikon) to observe cell lengths. The conidial suspension was dropped onto a hydrophobic coverslip and incubated in a dark moist chamber at 28°C for 0, 2, 4, 6, 8, 12, 18, and 24 hpi, then treated with KI/I_2_ solution (60 mg/ml KI, 10 mg/ml I_2_) or Nile red solution (50 mm Tris/maleate buffer, 20 mg/ml polyvinylpyrrolidone, 2.5 μg/ml Nile red, pH 7.5) to stain the glycogen and lipids. For mycelial vacuole staining, mycelia were treated with 10 μM 7‐amino‐4‐chloromethylcoumarin (CMAC; Thermo Fisher Scientific) for 30 min.

### 4.4 RT‐qPCR analysis

To evaluate the expression profile of *UBP3*, tissues of mycelia incubated in liquid CM, conidia produced on OTA plates, appressoria formed on the hydrophobic surface at 3 and 12 hpi, and infection hyphae formed in barley epidermal cells at 18, 24, and 48 hpi, were harvested to extract total RNA for preparing the cDNA templates. The RT‐qPCR experiment was performed using an SYBR Green PCR Master Mix kit (Takara) on an ABI 7500 real‐time PCR system (Applied Biosystems).

### 4.5 Yeast two‐hybrid assays

The cDNA of tested genes was cloned into yeast expression prey vector pGADT7 or yeast expression vector pGBKT7. The AD and BD combination vectors were co‐transformed into the yeast strain AH109 (Clontech). The vectors BD‐53/AD‐T and BD‐Lam/AD‐T were expressed in yeast as positive and negative controls, respectively. Log‐phase cultures of AH109 yeast cells containing plasmids carrying different combinations of prey (AD) and bait (BD) vectors were grown on selective (SD−Leu−Trp−His) plates and incubated at 30°C for 3 days. Aureobasidin A (AbA; Sigma‐Aldrich) was added to the −Leu−Trp−His plates to inhibit autoactivation. *LacZ* autoactivation was observed when Smo1 was cloned in the BD vector and Ubp3 was cloned in the AD vector. The solution was spread on the SD−Leu−Trp and SD−Leu−Trp−His plates, which then were cultured at 30°C for 3 days.

### 4.6 Co‐IP assay

The pKNRG‐*UBP3* and pKNFLAG‐*SMO1* vectors under the control of the constitutive RP27 promoter (Table S2) were constructed to confirm the interaction between Ubp3 and Smo1, which were co‐transformed into P131. The subsequent transformant WT/Ubp3‐Smo1 was incubated in 200 ml of liquid CM by inoculating mycelial fragments, then spread on an OTA plate (5 dpi). After incubation on a shaking incubator at 150 rpm for 48 h at 28°C, 1 g of fresh mycelia was harvested and washed with sterile water. The mycelia were ground in liquid nitrogen and used for extraction of total protein in the extraction buffer (10 mM Tris–HCl [pH 7.5], 150 mM NaCl, 0.5 mM EDTA, 0.5% Triton X‐100, 1 mM PMSF, protease inhibitor cocktail [Sigma]). Total proteins were then incubated at room temperature for 2 h after addition of 20 µl of anti‐GFP beads (Bimake) to capture the FLAG:Smo1 protein complexes. The beads were washed three times in PBST (phosphate‐buffered saline with Tween 20) buffer and three times in 50 mM TMAB (trimethylammonium bicarbonate, pH 8.5) buffer, then immunoprecipitated proteins were eluted off the beads by adding 100 µl of elution buffer (0.1% RapiGest in 50 mM TMAB). Subsequently, the immunoprecipitated proteins were boiled for 5 min for SDS‐PAGE assay. Western blotting was performed with anti‐GFP antibody (1:5000; Yeasen) or anti‐FLAG antibody (1:5000; Yeasen). The blotting signals were detected with X‐ray films (Fuji).

### 4.7 Western blotting

For protein extraction, around 0.2 g of mycelia was ground into powder in liquid nitrogen and resuspended in 1 ml of extraction buffer (10 mM Tris‐HCl [pH 7.5], 150 mM NaCl, 0.5 mM EDTA, 0.5% Triton X‐100) added with 1 mM PMSF (Sigma‐Aldrich). Total proteins were separated on 12% SDS‐PAGE gel for western blotting, and anti‐GFP (1:5000; Yeasen) was used as the primary antibody and anti‐mouse horseradish peroxidase (1:10,000; Yeasen) as the secondary antibody.

To determine the ubiquitination level of Smo1 in *M. oryzae*, proteins were extracted at different developmental stages and then incubated at room temperature for 2 h after addition of 20 µl of anti‐FLAG M2 beads (Bimake). The beads were washed three times in PBST and resuspended in 50 µl of protein loading buffer, then boiled for 5 min for SDS‐PAGE assay. Western blotting was performed with anti‐ubiquitin antibody (1:5000; PTMBIO).

## AUTHORS CONTRIBUTIONS

Y.N. and X.L.C. conceived the study. Y.N. and X.L.C. designed the experiments. X.C., Z.W., D.C. X.Z., W.H., and C.L. performed experiments. X.C., Y.N., Y.L., and X.L.C. analysed data and wrote the manuscript. Y.N., Y.L., X.L.C., and G.L. supervised the project. All authors discussed the results and contributed to the final manuscript.

## CONFLICT OF INTEREST

The authors declare no conflict of interest exists.

## Supporting information


**FIGURE S1** Western blots and quantitation of GFP:Rpl25 from the wild‐type P131 harvested at various times without rapamycin. GAPDH was used as an internal control

**FIGURE S2** Yeast two‐hybrid analysis confirmed protein–protein interactions between Ubp3 and Cdc48 (a), or between Ubp3 and Ufd3 (b). Cultures of AH109 yeast cells containing plasmids encoding different combination of prey (AD) and bait (BD) vectors were plated on selective (−Leu−Trp−His) plates and incubated at 30°C for 3 days. The vectors BD‐53/AD‐T and BD‐Lam/AD‐T were expressed in yeast as positive and negative controls, respectively. The cyclic depsipeptide antibiotic aureobasidin A (AbA) was added to the −Leu−Trp−Ade−His plate to inhibit autoactivation. *LacZ* autoactivation was observed when Cdc48 was cloned on the BD vector and Ubp3 was cloned on the AD vector

**TABLE S1** Fungal strains used in this study

**TABLE S2** Plasmids used in this study

## Data Availability

The data that support the findings of this study are available from the corresponding author upon reasonable request.
