# Supplementary material for: Deubiquitinase Ubp3 regulates ribophagy and deubiquitinates Smo1 for appressorium‐mediated infection by Magnaporthe oryzae
Source: Mol Plant Pathol. 2022 Feb 27;23(6):832–44. doi: 10.1111/mpp.13196 (PMC9104258; doi:10.1111/mpp.13196)
Supplement: Supplementary file 1 — FIGURE S1 Western blots and quantitation of GFP:Rpl25 from the wild‐type P131 harvested at various times without rapamycin. GAPDH was used as an internal control [file MPP-23-832-s003.pdf]

Figure S1

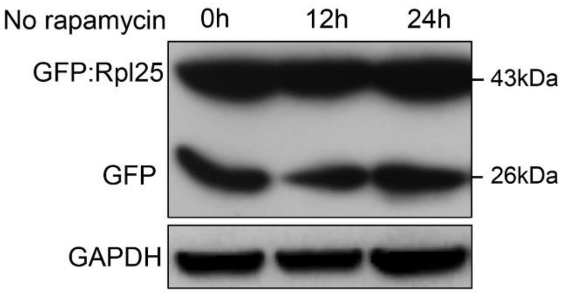

Fig. S1. Western blots and quantitation of GFP:Rpl25 from the wild type P131 harvested at various times without rapamycin. The GAPDH was used as an internal control.
