# Supplementary material for: Deubiquitinase Ubp3 regulates ribophagy and deubiquitinates Smo1 for appressorium‐mediated infection by Magnaporthe oryzae
Source: Mol Plant Pathol. 2022 Feb 27;23(6):832–44. doi: 10.1111/mpp.13196 (PMC9104258; doi:10.1111/mpp.13196)
Supplement: Supplementary file 2 — FIGURE S2 Yeast two‐hybrid analysis confirmed protein–protein interactions between Ubp3 and Cdc48 (a), or between Ubp3 and Ufd3 (b). Cultures of AH109 yeast cells containing plasmids encoding different combination of prey (AD) and bait (BD) vectors were plated on selective (−Leu−Trp−His) plates and incubated at 30°C for 3 days. The vectors BD‐53/AD‐T and BD‐Lam/AD‐T were expressed in yeast as positive and negative controls, respectively. The cyclic depsipeptide antibiotic aureobasidin A (AbA) was added to the −Leu−Trp−Ade−His plate to inhibit autoactivation. LacZ autoactivation was observed when Cdc48 was cloned on the BD vector and Ubp3 was cloned on the AD vector [file MPP-23-832-s001.pdf]

Figure S2

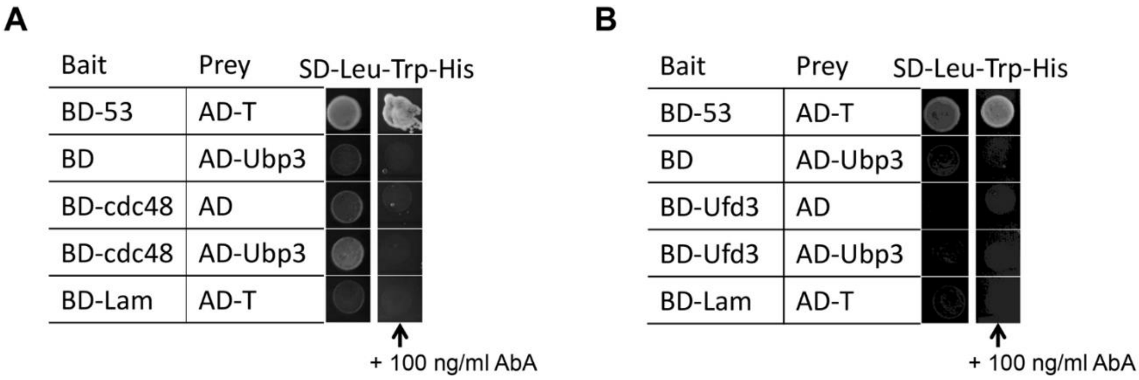

Fig. S2. Yeast two-hybrid analysis confirmed protein–protein interactions between Ubp3 and Cdc48 (A), or between Ubp3 and Ufd3. Cultures of AH109 yeast cells containing plasmids encoding different combination of prey (AD) and bait (BD) vectors were plated selective (-Leu-Trp-His) plates and incubated at 30 °C for 3 days. The vectors BD-53/AD-T and BD-Lam/AD-T were expressed in yeast as positive and negative controls, respectively. A cyclic depsipeptide antibiotic Aureobasidin A (AbA) were added in the -Leu-Trp-His plate to inhibit autoactivation. LacZ autoactivation was observed when Cdc48 was cloned on the BD vector and Ubp3 was cloned on the AD vector.
