# Supplementary material for: Deubiquitinase Ubp3 regulates ribophagy and deubiquitinates Smo1 for appressorium‐mediated infection by Magnaporthe oryzae
Source: Mol Plant Pathol. 2022 Feb 27;23(6):832–44. doi: 10.1111/mpp.13196 (PMC9104258; doi:10.1111/mpp.13196)
Supplement: Supplementary file 3 — TABLE S1 Fungal strains used in this study [file MPP-23-832-s002.docx]

**Table S1 Fungal strains used in this study.**

| **Strains** | **Genotypes** | **References** |
| --- | --- | --- |
| P131 | A wild-type isolate of *M. oryzae* | Peng and Shishiyama (1988) |
| Δ*ubp3-1*, Δ*ubp3-2* | *UBP3* deletion mutants of P131, Δ*ubp3*. | This study |
| cUBP3 | Complement strain of Δ*ubp3*, Δ*ubp3/UBP3* | This study |
| WT/GFP:Rpl25 | P131 transformed by *eGFP-RPL25* fusion construct, WT/GFP: RPL25. | This study |
| WT/GFP:Atg8 | P131 transformed by *eGFP-ATG8* fusion construct, WT/GFP:ATG8. | This study |
| ubp3/GFP:Rpl25 | Δ*ubp3* transformed by *eGFP-RPL25* fusion construct, Δ*ubp3*/GFP: RPL25. | This study |
| ubp3/GFP:Atg8 | Δ*ubp3* transformed by *eGFP-ATG8* fusion construct, Δ*ubp3*/GFP: ATG8. | This study |
| WT/FLAG:Smo1 | P131 transformed by *FLAG-SMO1* fusion construct, WT/FLAG: SMO1. | This study |
| ubp3/FLAG:Smo1 | Δ*ubp3* transformed by *FLAG-SMO1* fusion construct, WT/FLAG: SMO1. | This study |
| WT/GFP:Ubp3 | P131 transformed by *eGFP-UBP3* fusion construct, WT/GFP:UBP3. | This study |
| WT/Ubp3-Smo1 | P131 co-transformed by *eGFP-UBP3* and *FLAG-SMO1* transformants. | This study |

Peng, Y.-L., and Shishiyama, J. (1988). Temporal sequence of cytological events in rice leaves infected with *Pyricularia oryzae. Can. J. Bot*. 66, 730–735.
