# Supplementary material for: Deubiquitinase Ubp3 regulates ribophagy and deubiquitinates Smo1 for appressorium‐mediated infection by Magnaporthe oryzae
Source: Mol Plant Pathol. 2022 Feb 27;23(6):832–44. doi: 10.1111/mpp.13196 (PMC9104258; doi:10.1111/mpp.13196)
Supplement: Supplementary file 4 — TABLE S2 Plasmids used in this study [file MPP-23-832-s004.docx]

**Table S2 Plasmids used in this study.**

| **Names** | **Descriptions** |
| --- | --- |
| pKN | Vector used to construct complementation vectors and other vectors; with the *NPTII* gene as a selective marker inserted into pKS^+^ (Yang et al., 2010). |
| pKN-*UBP3* | *UBP3* complementation vector; *UBP3* gene containing 1.5 kb promoter and 0.5 kb terminator regions were amplified and inserted into pKN. |
| pKNRG | Vector used to construct vectors to constitutively express selected genes; with the fungal constitutive promoter RP27 (Yang et al., 2010). |
| pKNRG-*UBP3* | Vector for sub-cellular localization of UBP3 protein; coding region of *UBP3* was cloned into vector pKNRG. |
| pKNRG-*RPL25* | Vector for sub-cellular localization of RPL25 protein; coding region of *RPL25* was cloned into vector pKNRG. |
| pKNRG-*ATG8* | Vector for sub-cellular localization of ATG8 protein; coding region of *ATG8* was cloned into vector pKNRG. |
| pKNFLAG-*SMO1* | Vector for ubiquitination of SMO1 protein; coding region of *SMO1* was cloned into vector pKNFLAG. |

Yang, J., Zhao, X., Sun, J., Kang, Z., Ding, S., Xu, J. R., & Peng, Y. L. (2010). A novel protein Com1 is required for normal conidium morphology and full virulence in Magnaporthe oryzae. Molecular plant-microbe interactions : MPMI, 23(1), 112–123.
